# Study protocol for prognosis and treatment strategy of peripheral persistent avascular retina after intravitreal anti-VEGF therapy in retinopathy of prematurity

**DOI:** 10.1186/s13063-020-04371-6

**Published:** 2020-06-08

**Authors:** Ying Yu, Jianxun Wang, Feng Chen, Wensi Chen, Nan Jiang, Daoman Xiang

**Affiliations:** Department of Ophthalmology, Guangzhou Women and Children’s Medical Center, Guangzhou Medical University, Guangzhou, 510230 China

**Keywords:** Retinopathy of prematurity, Anti-VEGF, Photocoagulation, Fundus fluorescence angiography, Prophylactic treatment

## Abstract

**Background:**

Prophylactic peripheral photocoagulation has been proposed to be applied to persistent, peripheral, avascular retina for retinopathy of prematurity (ROP) patients who have received an intravitreal injection of anti-vascular endothelial growth factor (anti-VEGF) agents. However, there are doubts regarding the necessity of this prophylactic action regardless of fundus fluorescein angiography (FFA) results. The adverse prognosis for persistent avascular retina after anti-VEGF therapy in ROP patients is not well understood. The relationship between vascular leakage and an adverse prognosis is also unknown. Therefore, it would be of value to study the above issues to shape the treatment strategy of persistent avascular retina after intravitreal anti-VEGF therapy in ROP patients.

**Methods/design:**

This is a prospective study of ROP patients with persistent avascular retina who have received anti-VEGF intravitreal therapy and have never received laser therapy. All the eyes being studied will be followed up and examined by FFA after anti-VEGF treatment and categorized into two groups, a leakage group and a non-leakage group according to the extent of vascular leakage from FFA results. The eyes being studied in the leakage group will be further randomized into two groups, a laser group and a non-laser group. A cohort study (observational) will be conducted on the non-leakage group and the non-laser group (with leakage) to investigate the incidence of an adverse prognosis for reactivation, retinal tear and retinal detachment; as well as to investigate the relationship between vascular leakage from FFA results and the abovementioned pathological changes. A randomized controlled study (experimental) will be conducted on the leakage group to compare the prognosis between the laser group and the non-laser group.

**Discussion:**

The present study aims to investigate the occurrence rates of an adverse prognosis including reactivation, retinal tear and retinal detachment after anti-VEGF therapy in ROP patients with persistent avascular retina; to assess the relationship between vascular leakage from FFA results and the abovementioned pathological changes; to compare the prognosis of persistent avascular retina with or without prophylactic peripheral photocoagulation in these patients; to shape the treatment strategy and provide evidence for the indications of prophylactic photocoagulation.

**Trial registration:**

Chinese Clinical Trial Registry (ChiCTR), ID: ChiCTR-ROC-17013253. Registered on 5 November 2017. http://www.chictr.org.cn/showproj.aspx?proj=22703

## Background

Retinopathy of prematurity (ROP) is the leading cause of childhood blindness around the world [1]. Routine therapy for ROP has changed from cryotherapy in the 1980s to laser therapy in 1990s [2–4]. More recently, anti-vascular endothelial growth factor (anti-VEGF) therapy has become a common treatment, as VEGF plays an important role in the pathogenesis of ROP [5].

Since 2007, intravitreal injection of anti-VEGF agents has been applied in ROP treatment alone or in combination with laser therapy or vitrectomy [6]. However, some cases of ROP reactivation after this therapy have been reported, which could lead to retinal tear or detachment [7–10]. In comparison, laser treatment is a therapy that could permanently inhibit peripheral vascularization [5, 11]. Thus, it has been proposed to apply prophylactic peripheral photocoagulation to persistent avascular retina for ROP patients who have received intravitreal injection of anti-VEGF agents [12]. However, there are doubts regarding the necessity of this prophylactic action, as laser therapy is a destructive treatment and, notably, some studies have demonstrated that although vascularization is incomplete after anti-VEGF therapy in some cases, none of these cases have developed pathological neovascularization or complications [13–15].

In addition, it has been reported that vascular leakage exists in around half of the eyes treated with anti-VEGF therapy, which could lead to subsequent retinal tear or detachment in some cases [15]. Hence, some articles have claimed that vascular leakage could be a sign of disease persistence [6] and the most significant sign of progression to severe and surgical ROP [16]. However, there is also a lack of evidence as to whether the presence of vascular leakage and/or the leakage amount is actually related to an adverse prognosis or not. Fundus fluorescein angiography (FFA) imaging is a valuable diagnostic tool which can detect some ocular lesions in early phases, including vascular leakage [17]. If the presence of vascular leakage and/or the leakage amount is indeed a predictor of an adverse prognosis, it is possible that by applying FFA, we could reach a more accurate way of judging treatment selection, taking the extent of vascular leakage into consideration.

In all, the incidence of adverse prognosis for persistent avascular retina after anti-VEGF therapy in ROP patients is not well understood. The relationship between vascular leakage and an adverse prognosis is also unknown. However, laser therapy could inevitably cause permanent damage to peripheral visual function and induce significant myopia. Therefore, it is necessary to study the incidence and mechanism of an adverse prognosis of persistent avascular retina after anti-VEGF therapy in ROP patients based on FFA of the peripheral retina, including the ora serrata region, in order to shape the treatment strategy and provide evidence for the indications of prophylactic photocoagulation.

## Methods/design

### Aim

To investigate the occurrence rates of an adverse prognosis, including reactivation, retinal tear and retinal detachment after anti-VEGF therapy, in ROP patients with persistent avascular retina; to assess the relationship between vascular leakage from FFA results and the abovementioned pathological changes; to compare the prognosis of persistent avascular retina with or without prophylactic peripheral photocoagulation in these patients; to shape the treatment strategy and provide evidence for the indications of prophylactic photocoagulation.

### Study design

**Fig. 1:**
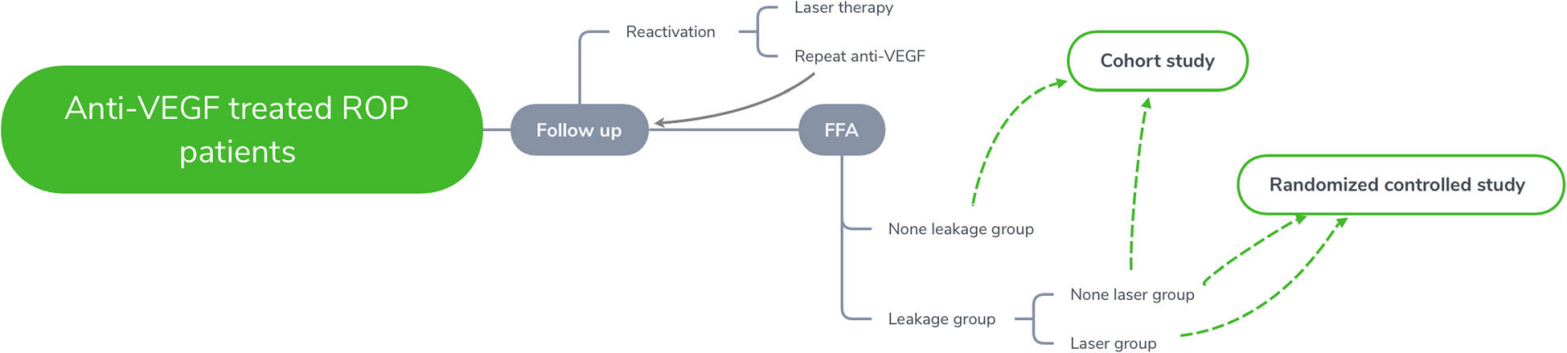
Study design and grouping methods

This is a prospective, simple, parallel, randomized study of ROP patients with persistent avascular retina who have received anti-VEGF intravitreal therapy and have never received laser therapy. All the eyes studied will be followed up and examined by FFA after anti-VEGF treatment and categorized into two groups, a leakage group and a non-leakage group according to the presence/extent of vascular leakage from the FFA results. The eyes studied in the leakage group will be further randomized into two groups, a laser group and a non-laser group (Fig. 1). A cohort study (observational) will be conducted to compare between the non-leakage group and the non-laser group (with leakage); a randomized controlled study (experimental) will be conducted to compare between the laser group and the non-laser group.

### Outcomes

**Fig. 2:**
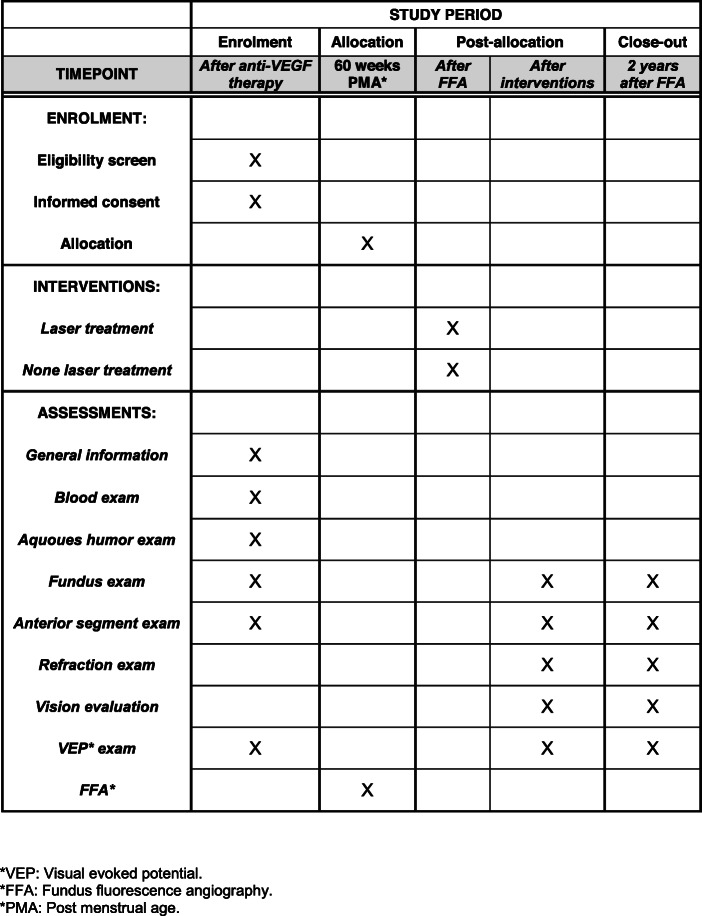
Schedule of enrollment, interventions and assessments. *VEP visual-evoked potential, *FFA fundus fluorescence angiography, *PMA post-menstrual age

Between the non-leakage group and the non-laser group, and also between the laser group and the non-laser group, the following outcomes will be compared. For the primary outcomes, the incidence of reactivation, retinal tear and retinal detachment will be calculated in all cases and compared using the chi-square test (binary variables). For the secondary outcomes: (1) refraction, vision, visual-evoked potential (VEP) level, the peripheral non-perfusion area quantified from the vascular termination zone to the ora serrata, and the timing of regression, reactivation, stabilization as well as complete vascularization will be compared using Student’s *t* test or the Mann-Whitney *U* test (continuous variables); (2) the relationship between vascular leakage from the FFA results and the abovementioned variables will be tested using Spearman’s correlation test (Fig. 2).

Reactivation is defined as the redevelopment of extra-retinal fibrovascular proliferation, whether due to re-growth or at another more anterior location, after initial regression. Stabilization is defined as the existence of an avascular area with a boundary from the vascular area, without neovascularization or fibrogenesis, or dilated and tortuous vessels at the posterior pole. Completed vascularization is defined as vascular termini within a 1.5-disk diameter of the temporal ora serrata and a 0.5-disk diameter of the nasal ora serrata [12].

### Study setting

ROP patients hospitalized in the Department of Ophthalmology, Guangzhou Women and Children’s Medical Center, Guangzhou Medical University, Guangzhou, China during the time span from January 2020 to January 2022 will be considered for enrollment.

The sample size was calculated using http://powerandsamplesize.com (USA) [18]. We estimate that the rate of reactivation, retinal tear and retinal detachment in the non-leakage group and the laser group (with leakage) would both be very close to zero, which could be inputted as 0.001. Also, because it was reported that the recurrence incidence after anti-VEGF intravitreal therapy was 8.3% in 251 premature infants with type-1 ROP [19], we estimate that the rate of reactivation, retinal tear and retinal detachment in the non-laser group (with leakage) would be around 0.100 (higher than 0.083 because of leakage).

Using 0.001 and 0.100 as event rates, a 5% type-1 error, and a power of 80%, the needed sample size would be 73 cases. Therefore, the required sample size for comparison of the non-leakage group vs. the laser group, and the comparison of the laser group vs. the non-laser group would both be 73 cases. The needed sample size for the whole study would be 110 cases. Under preliminary estimation, the included case number will be around 120–170 cases.

We will publish the information about trial recruitment on the official platform of our hospital in a nationally, widely used social app named Wechat (China). Those who enter the trial will have the privilege of priority in follow-up and medical consulting, as a strategy for achieving adequate participant enrollment to reach target sample size.

### Inclusion criteria

ROP patients with persistent avascular retina who have received anti-VEGF therapy (once or having multiple episodes of standard intravitreal injection of 0.03 mg Lucentis at a site of 0.5 mm to 1.0 mm from the retinal limbus under general anesthesia) and agree to participate will be included. The post-menstrual age (PMA) of the included ROP patients will be < 60 weeks. Persistent avascular retina is defined as an observed avascular retina by 60 weeks of PMA. The requirement of anti-VEGF therapy is a diagnosis of threshold ROP or pre-threshold type-I ROP, as well as of aggressive posterior ROP (APROP). APROP is a severe form of ROP that affects posterior location (zone I or posterior zone II), characterized by a flat neovascular network at the simple junction between vascularized and nonvascularized retina, with increased dilation and tortuosity of retinal vessels in all four quadrants and intraretinal shunting.

ROP could be classified by stage, zone and the presence of plus disease [3]. The stage of ROP is defined by the appearance of the vessels at the interface between the avascular and the vascular retinal zones. Threshold ROP is stage-3 ROP in zones I or II with plus disease and with at least five contiguous or eight cumulative sectors (clock hours) of lesions [20]. Pre-threshold ROP is any stage of ROP in zone I that was less than the threshold ROP, or stage-2 ROP in zone II with plus disease, or stage-3 ROP in zone II without plus disease, with five contiguous or eight cumulative clock hours of lesions [20]. APROP is ROP with significant plus disease and an ill-defined form of the retinopathy located in the posterior retina [21].

### Exclusion criteria

Patients who have received laser therapy before FFA is performed; patients with an opacity in the refracting media that could prevent staging; and patients with contraindications for general anesthesia or FFA will be excluded.

### Randomization

Opaque, sealed envelopes will be used for the intervention assignments after verification of eligibility. A clinician who is not in the trial group will prepare two envelopes, one for the laser group and one for non-laser group, and give the envelopes to one of the clinicians in the trial group who will open one of the envelopes. Infants born from multiple gestations will be assigned individually.

### Trial intervention

Zero point five milliliters of aqueous humor and 5-mL blood samples will be collected from all patients after they are included in this study. After anti-VEGF treatment, they will be followed up weekly for 1 month, biweekly for 2 months and then every 3–4 weeks until stabilized and/or laser therapy is performed

FFA will be performed under general anesthesia using RetCam (USA) on all the studied eyes once the patients reached 60 weeks of PMA, or sooner if there is reactivation. Then, all the studied eyes will be classified according to the FFA results as mentioned above. The laser group will be treated by Dr. Daoman Xiang with a 532 laser (200 mJ energy, 0.3-s interval time and 0.3-s duration pulse) using LUMENIS (USA) under general anesthesia. The extent of the laser treatment is all the leakage area and all the avascular area. The timing will be immediately after FFA reveals leakage. The non-laser group will only be observed. Then, all patients will be followed up for 2 years after FFA. This time period has been chosen because ROP reactivation/detachment events after intravitreal therapy are unlikely to happen more than 2.5 years after PMA [10, 12], and to improve the compliance for follow-up visits.

### Modification

During the follow-up observation before FFA is performed, the studied eyes with reactivation of ROP will be given repeated anti-VEGF therapy or laser therapy according to the outcome of the discussion with the treating physician and the participant’s family. Those who receive laser therapy at this point (before FFA) will be excluded from further intervention or follow-up, but will still be recorded. No other treatments will be allowed during the trial. If any other treatments were given, the patient will also be excluded from further intervention or follow-up, but these treatments will be recorded.

### Data collection

#### General information

General information of the patients will be collected, including gender, gestational weeks, whether single or multiple birth, PMA at onset, type and length of oxygen-inhalation history, Apgar scores, and systemic infection state.

#### Examination data

Examination data of the patients will be collected, including for the fundal, anterior segment, refraction, vision, VEP, blood and aqueous humor examination results. The fundus will be examined using RetCam (USA), except for the last fundal examination in this study (2 years after FFA), which will be performed with the Optos Scanning Laser Ophthalmoscope Model: California (P200DTx) (UK), with the patient being sedated. Fundal examination results will be recorded including the stage, zone and type of ROP, as well as the peripheral non-perfusion area quantified from the area of vascular termination to the ora serrata, the presence or not of plus disease, circumferential vessels, terminal bulbs, fibrovascular proliferation, retinal tear and retinal detachment. Aqueous humor examination includes the concentration of VEGF, pigment epithelial-derived factor (PEDF) and b fibroblast growth factor (bFGF). Blood examination includes routine and biochemistry tests, serum VEGF, PEDF and bFGF concentration tests.

#### Other information

Treatment information and time point of changes will also be collected. Treatment information includes the type and parameters of any treatment. The time point of changes includes the time point of regression, reactivation, stabilization and complete vascularization.

### Data management

The data analyst will be blinded when given the data (group names and patients’ information will be concealed) until the data are published. Microsoft Office Excel 2007 software will be used for data entry and storage. The data will be collected by one clinician and collated by another clinician who will also have the range checks for data values. Continuous variables will be examined three times to record the average levels. Cases with missing data will be excluded from statistical analysis.

### Statistical methods

SPSS 20.0 software will be applied for the statistical analysis. Parameters that comply with normal distribution will be evaluated by Student’s *t* test and a one-way analysis of variance (ANOVA) test. Pearson’s correlation test will be applied to analyze bivariate relationships. Parameters that do not comply with normal distribution will be evaluated by the Mann-Whitney *U* test and the Kruskal-Wallis test. Spearman’s correlation test will be applied to analyze bivariate relationships. The chi-square test will be applied to analyze categorical variables. Regression analysis will be applied to detect the significance of influential factors. *P* < 0.05 will be considered statistically significant.

### Trial Governance Committee

The Governance Committee will include: Prof. Daoman Xiang, Dr. Ying Yu, Dr. Jianxun Wang, Dr. Feng Chen, Dr. Wensi Chen, Dr. Nan Jiang, all from Department of Ophthalmology, Guangzhou Women and Children’s Medical Center, Guangzhou Medical University, Guangzhou, China. Prof. Daoman Xiang can make protocol decisions. Dr. Wensi Chen will be in charge of data collection. Dr. Feng Chen will be in charge of data collation. Dr. Nan Jiang will be in charge of data analysis. Dr. Jianxun Wang will be in charge of quality control. Dr. Ying Yu will be in charge of form development, database development and data management. All members will monitor the execution of the trial and will have access to the final trial dataset.

### Data Safety Monitoring Board

The Board members will collect, assess, report and manage solicited and spontaneously reported adverse events and other unintended effects of trial interventions or trial conduct. The Board will be independent from any competing interests. There is no intention to use professional writers. The Board members will include: Prof. Daoman Xiang, Dr. Ying Yu, Dr. Jianxun Wang, Dr. Feng Chen, Dr. Wensi Chen, Dr. Nan Jiang, all from the Department of Ophthalmology, Guangzhou Women and Children’s Medical Center, Guangzhou Medical University, Guangzhou, China. Prof. Daoman Xiang can make the final decision to terminate the trial. A clinician who is independent from the investigators and the sponsors will audit the trial conduct by random inspection once every month.

## Discussion

In premature infants, peripheral retinal vascularization is incomplete and vascularization will continue to develop after birth [22, 23]. After anti-VEGF therapy, peripheral avascular retina can still exist in some ROP patients. Hence, some studies have proposed to prophylactically treat these patients with peripheral photocoagulation in cases of persistent avascular retina to reduce the risk of complications like retinal tear and detachment [7–10, 12]. However, there are doubts regarding this prophylactic treatment, as the occurrence rates of retinal tear and detachment have been variedly reported [9, 24, 25] and laser therapy is a destructive treatment [5].

In addition, Tahija et al. found that in FFA results, vascular leakage existed in nine out of 20 anti-VEGF-treated eyes of infants [15]. Lepore et al. demonstrated that vascular leakage existed in 65% of eyes treated with anti-VEGF in patients at 4 years of age [6]. Some studies have claimed that the presence of vascular leakage is the sign of disease persistence [6] and the most significant sign of progression to severe ROP [16]. It is possible that certain components in the blood could induce secondary changes in the retina, leading to retinal tear and detachment. If so, there could be a much bigger risk of retinal tear and detachment in patients with vascular leakage found in FFA results than those without. However, this theory is also lacking in evidence.

In our experience, the vascular leakage of ROP is different from that of diabetic retinopathy. The pathological changes of diabetic retinopathy are active, and could continually develop. Without treatment, the retinal erosions caused by diabetes are likely to progress. However, for ROP cases, after anti-VEGF therapy, the development of blood vessels is usually suppressed from the initial state of random growth, without continuous stimulation.

Although the avascular zone persists in some patients, the retinal blood vessels usually cannot grow outside of a certain boundary, so they are anastomosed laterally along the boundary, forming a circular net. In these circumstances, vascular leakage may be unlikely to happen. And since the avascular retinal zone tends to be peripheral and usually does not reach the threshold phase or pre-threshold type-I ROP, prophylactic laser therapy could be an overtreatment for most cases, even for some cases with vascular leakage, let alone those without leakage. Also, there may be a necessity to take the amount of leakage into consideration when deciding whether or not to execute prophylactic laser treatment.

Herein, the present study aims to investigate the occurrence rates of an adverse prognosis including reactivation, retinal tear and retinal detachment after anti-VEGF therapy in ROP patients with persistent avascular retina; to assess the relationship between vascular leakage found in FFA results and the abovementioned pathological changes; to compare the prognosis of persistent avascular retina with or without prophylactic peripheral photocoagulation in these patients; to shape the treatment strategy and to provide evidence for the indications of prophylactic photocoagulation.

## Data Availability

The data and materials in this trial will be reserved by the trial group.

## Abbreviations

ROP: Retinopathy of prematurity
anti-VEGF: Anti-vascular endothelial growth factor
FFA: Fundus fluorescein angiography
APROP: Aggressive posterior retinopathy of prematurity
PMA: Post-menstrual age
PEDF: Pigment epithelial-derived factor
bFGF: B fibroblast growth factor
